# Improved growth performance, food efficiency, and lysine availability in growing rats fed with lysine-biofortified rice

**DOI:** 10.1038/s41598-017-01555-0

**Published:** 2017-05-02

**Authors:** Qing-Qing Yang, Pui Kit Suen, Chang-Quan Zhang, Wan Sheung Mak, Ming-Hong Gu, Qiao-Quan Liu, Samuel Sai-Ming Sun

**Affiliations:** 1 Key Laboratory of Crop Genetics and Physiology of Jiangsu Province/Key Laboratory of Plant Functional Genomics of the Ministry of Education, College of Agriculture, Yangzhou University, Yangzhou, 225009 China; 2 State Key Laboratory of Agrobiotechnology, School of Life Sciences, The Chinese University of Hong Kong, Shatin, Hong Kong China; 3 Co-Innovation Center for Modern Production Technology of Grain Crops of Jiangsu Province/Joint International Research Laboratory of Agriculture and Agri-Product Safety of the Ministry of Education, Yangzhou University, Yangzhou, 225009 China

**Keywords:** Biotechnology, Plant sciences

## Abstract

Rice is an excellent source of protein, and has an adequate balance of amino acids with the exception of the essential amino acid lysine. By using a combined enhancement of lysine synthesis and suppression of its catabolism, we had produced two transgenic rice lines HFL1 and HFL2 (High Free Lysine) containing high concentration of free lysine. In this study, a 70-day rat feeding study was conducted to assess the nutritional value of two transgenic lines as compared with either their wild type (WT) or the WT rice supplemented with different concentrations of L-lysine. The results revealed that animal performance, including body weight, food intake, and food efficiency, was greater in the HFL groups than in the WT group. Moreover, the HFL diets had increased protein apparent digestibility, protein efficiency ratio, and lysine availability than the WT diet. Based on the linear relationship between dietary L-lysine concentrations and animal performance, it indicated that the biological indexes of the HFL groups were similar or better than that of the WT20 group, which was supplemented with L-lysine concentrations similar to those present in the HFL diets. Therefore, lysine-biofortified rice contributed to improved growth performance, food efficiency, and lysine availability in growing rats.

## Introduction

Transgenic strategies may improve the nutritional value of crops and ensure food security^1^. Transgenic crops with enhanced nutritional value may have improved nutrient bioavailability and/or lower levels of anti-nutritional factors^2–4^, such as high-methionine lupin^5^, lupin that expresses methionine-rich sunflower albumin^6^, low-phytate transgenic maize^7^, and tryptophan-enriched rice^8^.

As an essential amino acid (EAA), lysine cannot be synthesized by humans or farm animals and represents an indicator of other dietary EAAs^9^. Animal growth performance, carcass characteristics, and immune function are affected by lysine deficiency^10–16^. Supplementation with synthetic lysine enhances nitrogen retention and protein accretion and improves animal growth performance and immune function^10, 17–20^. In cereals, lysine is a limiting EAA. In an attempt to reduce the incidence of lysine deficiency, cereals such as maize^21^, sorghum^22^, and rice^23–25^ have been biofortified with the amino acid. Animal studies have shown that the bioefficacy of high-lysine transgenic maize is similar to that wild-type maize supplemented with L-lysine, and high-lysine transgenic maize is considered to be more nutritious than wild-type maize^26–28^. In pigs, lysine bioefficacy is dependent on the source of dietary lysine^29^. Altogether, these studies have shown that animal weight gain, protein digestibility, and amino acid bioavailability are associated with increased dietary lysine.

Rice represents an important source of energy and protein for approximately one-third of the world’s population^30^. Rice has an adequate balance of amino acids with the exception of lysine^31^. We have developed several types of high-lysine transgenic rice by overexpressing endogenous rice histone proteins enriched with lysine^24^ or heterogeneous lysine-rich proteins^32^. Furthermore, we have generated high-lysine transgenic rice by modifying lysine metabolism^23, 25^. The transgenic rice lines HFL1 and HFL2 were developed by enhancing lysine anabolism and reducing lysine catabolism^25^. These two HFL transgenic lines contain free lysine levels in seeds up to 25-fold over that of the wild type, but without the selectable marker gene. All target transgenes in both transgenic lines were integrated into the intragenic region of the rice genome, with no differences in major agronomic traits, including yield^25^. These two transgenic rice lines, which are currently undergoing nutritional and food safety assessments, could potentially alleviate malnutrition^33^.

The objective of this study was to evaluate the nutritional value of HFL transgenic rice. Transgenic rice and their wild-type counterpart were fed to growing rats. Animal growth performance, food efficiency, and protein and amino acid availability were analyzed and compared. Additionally, to evaluate whether lysine in transgenic rice is bioavailable to animals, another feeding trial was performed with wild-type rice supplemented with different dosages of L-lysine (Table 1).

**Table 1:** Formulation of the experimental rice-based diets.

Ingredient (g/kg)	WT	WT10	WT20	WT40	HFL1	HFL2
Rice flour	849.5	849.3	849.0	848.5	849.5	849.5
L-Lysine	0	0.235	0.47	0.94	0	0
Corn oil	60	60	60	60	60	60
Fiber	40	40	40	40	40	40
Mineral mix (AIN-93G-MX)	35	35	35	35	35	35
Vitamin mix (AIN-93G-VX)	10	10	10	10	10	10
L-Cystine	3	3	3	3	3	3
Choline bitartrate	2.5	2.5	2.5	2.5	2.5	2.5
Tert-butylhydroquinone	0.014	0.014	0.014	0.014	0.014	0.014

## Results

### Grain composition and amino acid balance in HFL and WT rice

**Figure 1:**
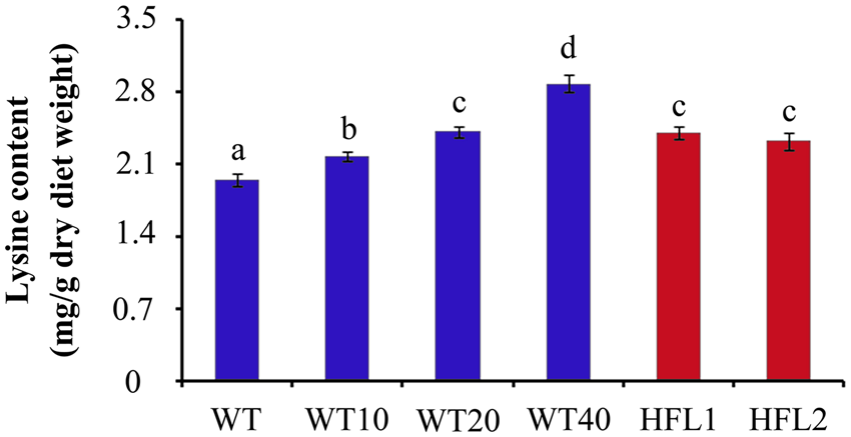
Lysine concentrations in the different diets. Error bars represent SD (n = 3). Different letters represent significant differences (*P* < 0.05).

The composition of HFL and WT rice grains is shown in Table 2. The free lysine level dramatically increased in the two HFL lines, and total lysine content was higher in HFL1 (+24%) and HFL2 (+19%) than in WT rice. Additionally, the total protein content in transgenic rice seeds was also slightly higher, by 1.05% and 0.85% in HFL1 and HFL2, respectively, than that of WT rice. There were no significant differences in moisture, ash, fiber, lipid, or carbohydrate contents between the transgenic and WT rice flour. Furthermore, in rat feeding study, the two HFL diets showed higher lysine content than the non-supplemented WT diet (Fig. 1 and Supplementary Table S1) as well.

**Table 2:** Grain composition of HFL and wild type rice (n = 3).

Composition*	WT	HFL1	HFL2
Free lysine (μg/g)	20.91 ± 0.93 a	427.89 ± 22.55 b	314.59 ± 3.38 c
Total lysine (mg/g)	2.35 ± 0.01 a	2.89 ± 0.02c	2.77 ± 0.02 b
Protein (%)	6.74 ± 0.07 a	7.79 ± 0.08 b	7.59 ± 0.08 b
Fiber (%)	0.39 ± 0.10 a	0.32 ± 0.02 a	0.30 ± 0.00 a
Fat (%)	0.70 ± 0.06 a	0.66 ± 0.06 a	0.76 ± 0.06 a
Ash (%)	0.71 ± 0.05 a	0.65 ± 0.04 a	0.63 ± 0.04 a
Carbohydrate (%)	87.03 ± 0.94 a	86.13 ± 1.75 a	86.94 ± 2.09 a
Moisture (%)^*^	5.26 ± 0.52 a	5.92 ± 0.93 a	5.22 ± 0.80 a

*Dry weight basis except for moisture. Different letters represent significant differences from WT (*P* < 0.05).

We calculated the amino acid scores (AAS) in HFL and WT diets (Supplementary Table S2) based on the protein reference pattern recommended for school-aged children and adolescents^34^. In WT rice, the first, second, and third limiting EAAs are lysine, threonine, and leucine, respectively^35^. In this study, the WT diet had a total lysine content of 1.94 mg/g (Fig. 1) and an AAS of 0.75 (Supplementary Table S2). HFL1 and HFL2 had a total lysine content of 2.40 and 2.32 mg/g, respectively (Fig. 1), and an AAS of 0.83 and 0.78, respectively (Supplementary Table S2). Lysine was the first limiting EAA in transgenic and WT rice. However, lysine AAS was significantly higher in the two transgenic diets than in the WT diet. There were no differences in threonine and leucine AAS between the transgenic and WT diets (Supplementary Table S2). Therefore, diets containing transgenic rice HFL1 and HFL2 had a better amino acid balance than diets containing WT rice.

### Enhanced growth performance in HFL groups

**Figure 2:**
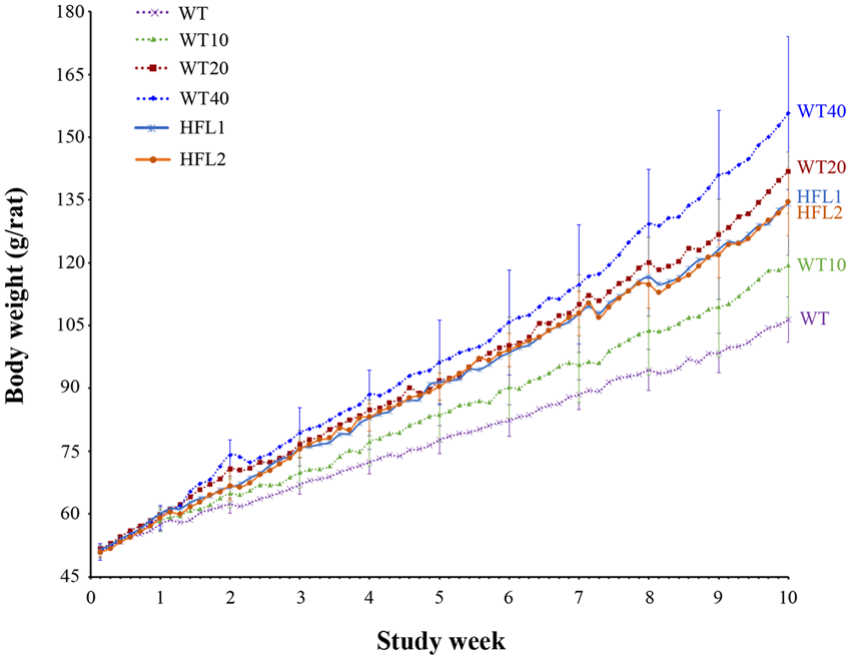
Body weight changes in rats fed different diets for 70 d.

During the 70-d feeding trial, the rats grew well, and no diseases or deaths were recorded. Body weight changes are presented in Fig. 2 and Supplementary Fig. S1. All rats, which had a similar initial body weight of ~50 g (Supplementary Table S2), grew similarly during the preliminary feeding stage (the first 7-d). Following this preliminary feeding stage, rats fed ad libitum either HFL transgenic rice diets or L-lysine-supplemented WT rice diets grew faster than those fed the non-supplemented WT rice diet. Interestingly, body weight increased with increasing lysine concentrations (from 1.94 mg/g in WT to 2.88 mg/g in WT40; Fig. 2 and Supplementary Table S3).

Compared with the WT group, the HFL groups had greater growth performance (Fig. 2). At the end of 70-d feeding trial, the final body weights of the HFL1 and HFL2 groups were 134.08 g/rat and 134.54 g/rat, respectively, which were 25.97% and 26.40% higher, respectively, than that of the WT group (Supplementary Table S3). The growth curve of the two HFL groups was quite similar to that of the WT20 group. The WT20 diet contained 20% more L-lysine than the WT diet and similar total lysine concentrations as the HFL diets (Fig. 1).

### Increased food efficiency in rats fed with HFL rice diets

**Figure 3:**
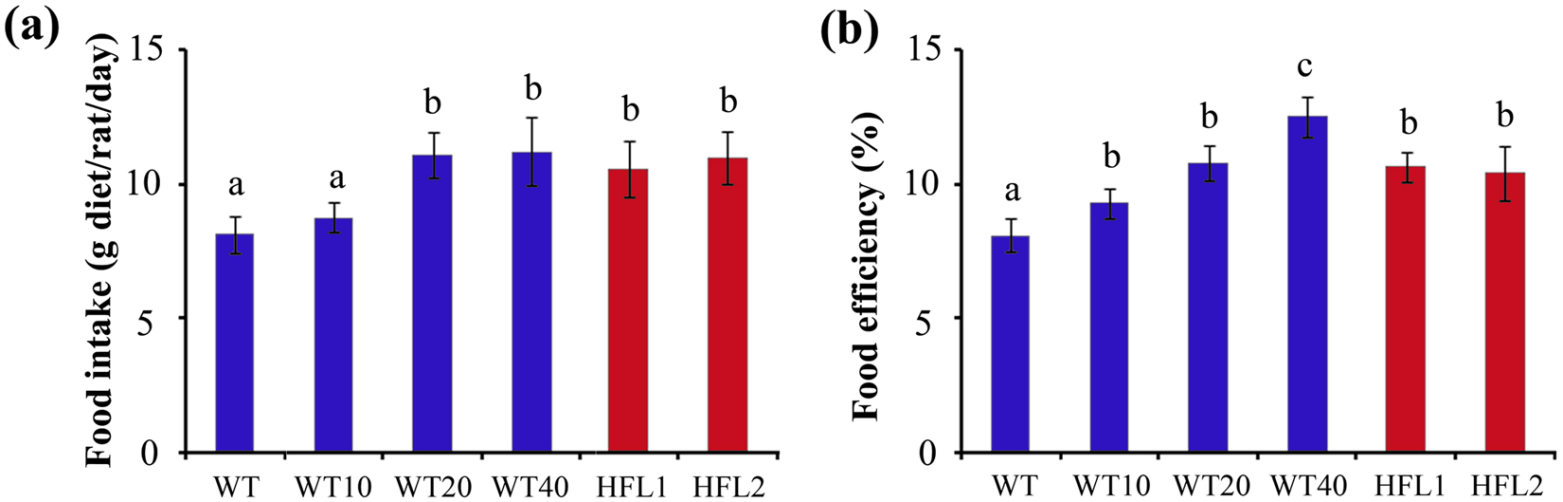
Food intake (**a**) and food efficiency (**b**) of rats fed different diets for 70 d. Error bars represent SD (n = 8). Different letters represent significant differences (*P* < 0.05).

After one week of preliminary feeding, the food intake, body weight gain and food efficiency increased in either HFL or L-lysine-supplemented WT groups than in WT group. At the end of the experiment, the HFL groups had higher food intake and food efficiency than the WT group (Fig. 3 and Supplementary Table S3). Similarly, food intake, body weight gain and food efficiency were higher in the L-lysine- supplemented groups than in the WT group (Fig. 3). Food efficiency of the HFL1 and HFL2 diets was similar to that of the WT10 and WT20 diets, but lower than that of the WT40 diet.

### Improved protein digestibility and nitrogen balance in HFL groups

**Figure 4:**
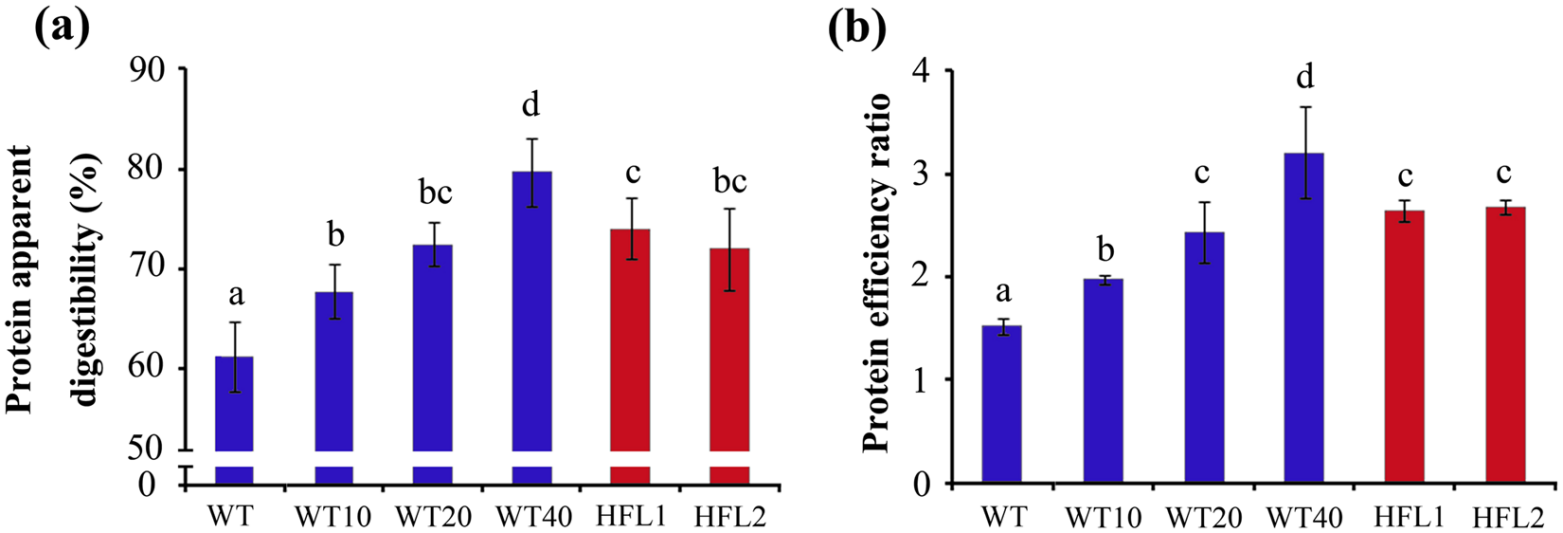
Protein apparent digestibility (**a**) and protein efficiency ratio (**b**) of rats fed different diets. Error bars represent SD (n = 8). Different letters represent significant differences (*P* < 0.05).

During the nitrogen-balance stage (6-d following the 7-d acclimation period), fecal and urine samples were collected separately and analyzed. There were no significant differences in total feces (dry weight, g/6 d/rat) or fecal nitrogen (g/6 d/rat) among the six groups (Supplementary Fig. S2). Therefore, fecal excretion was normal during the experimental stage. The HFL1 and HFL2 groups had higher total protein intake (Supplementary Fig. S2) and greater protein apparent digestibility than the WT group (Fig. 4a). The protein efficiency ratio (PER) of the HFL groups was higher than that of the WT group, but similar to that of the WT20 group (Fig. 4b).

Among the L-lysine-supplemented WT diets, their protein apparent digestibility reached highest in the case of WT40 while improvement was observed accordingly with increasing lysine content in the diet from 1.94 mg/g to 2.88 mg/g (Fig. 4a). In addition, the PER was also significantly enhanced in accordance with the increase in supplementary L-lysine in the diets between the WT10 and WT40 groups (Fig. 4b).

### Improved availability of EAAs in growing rats fed HFL rice diets

**Figure 5:**
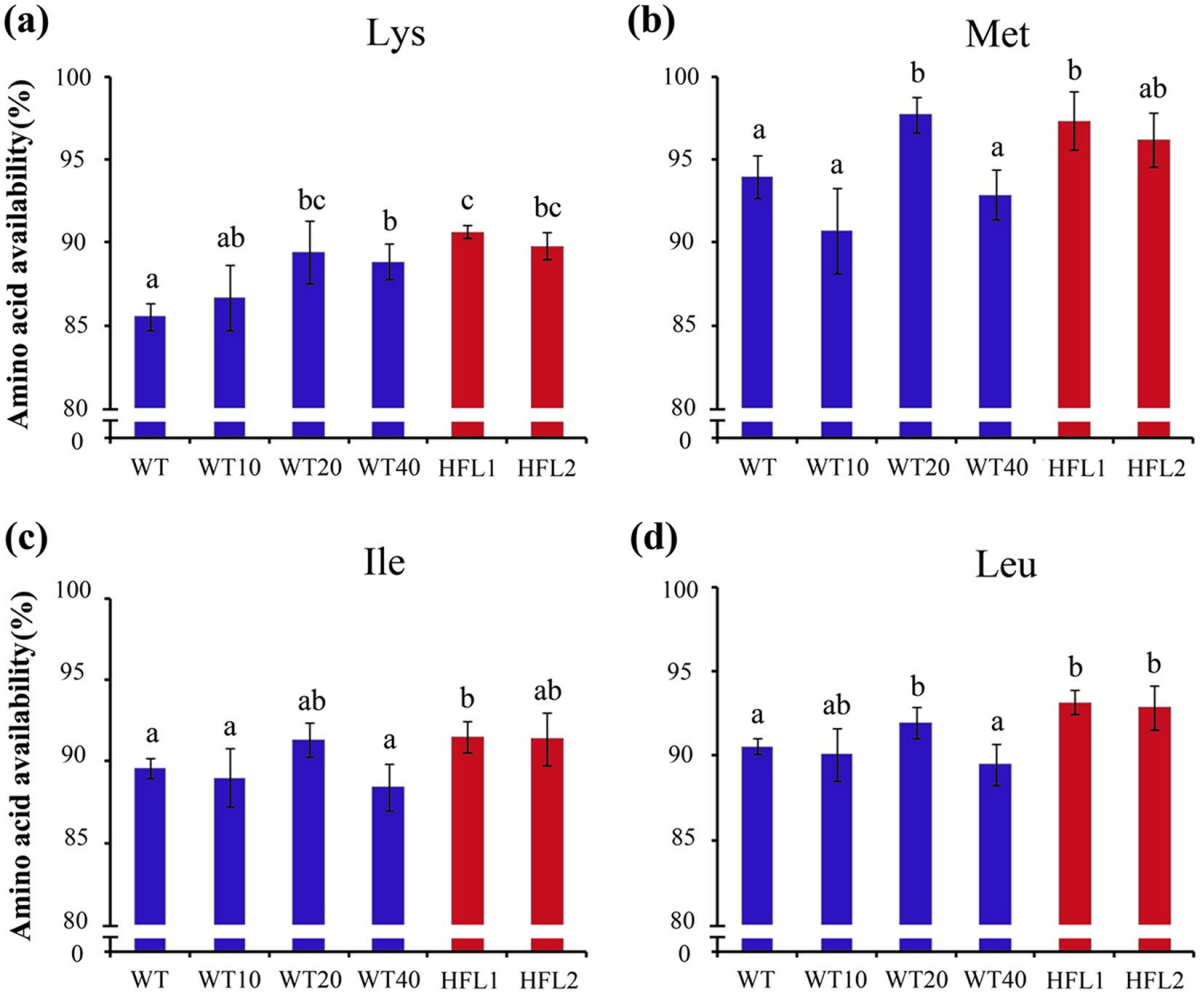
Availability of essential amino acids lysine (**a**), methionine (**b**), isoleucine (**c**), and leucine (**d**) in rats fed different diets. Error bars represent SD (n = 8). Different letters represent significant differences (*P* < 0.05).

The availability of most amino acids is presented in Fig. 5 and Supplementary Fig. S3. As expected, lysine availability was 90.69% and 89.85% for the HFL1 and HFL2 groups, respectively, which was higher than that of the WT or L-lysine-supplemented WT groups, especially of the WT group (85.57%; Fig. 5a). Among the L-lysine-supplemented groups, lysine availability was the highest in the WT20 group, but not in others including WT 40, implying that there is an optimum requirement of lysine level in the diet for animal feeding.

In addition to lysine, leucine and threonine are also limiting EAAs in rice. Even though the AAS of lysine was higher in the two HFL transgenic rice diets than in the WT diet, the AAS of leucine and threonine were similar between the transgenic HFL and WT diets. Interestingly, the availability of other three EAAs, including leucine, methionine and isoleucine, improved in HFL rice diets (Fig. 5b–d). There was no difference in the availability of other amino acids between transgenic HFL and WT diets (Supplementary Fig. S3).

### Effect of HFL diets on viscera coefficient

At the end of the feeding experiment, the rats were anaesthetized and their main organs were collected and weighed. No abnormity was observed. The viscera coefficient of each group was calculated as the ratio between organ weight and body weight (Supplementary Table S4). There were no significant differences in viscera coefficient among the six groups. Liver and kidney coefficients of the HFL groups were lower than those of the WT group, but similar to those of the group consuming commercially available the standard rat diet (2018SX, Teklad Global, USA, containing 18% protein) (data no shown). Therefore, lysine had no effect on the health of the growing rats.

### Enhanced levels of serum lysine and muscle nitrogen in growing rats fed HFL diets

**Figure 6:**
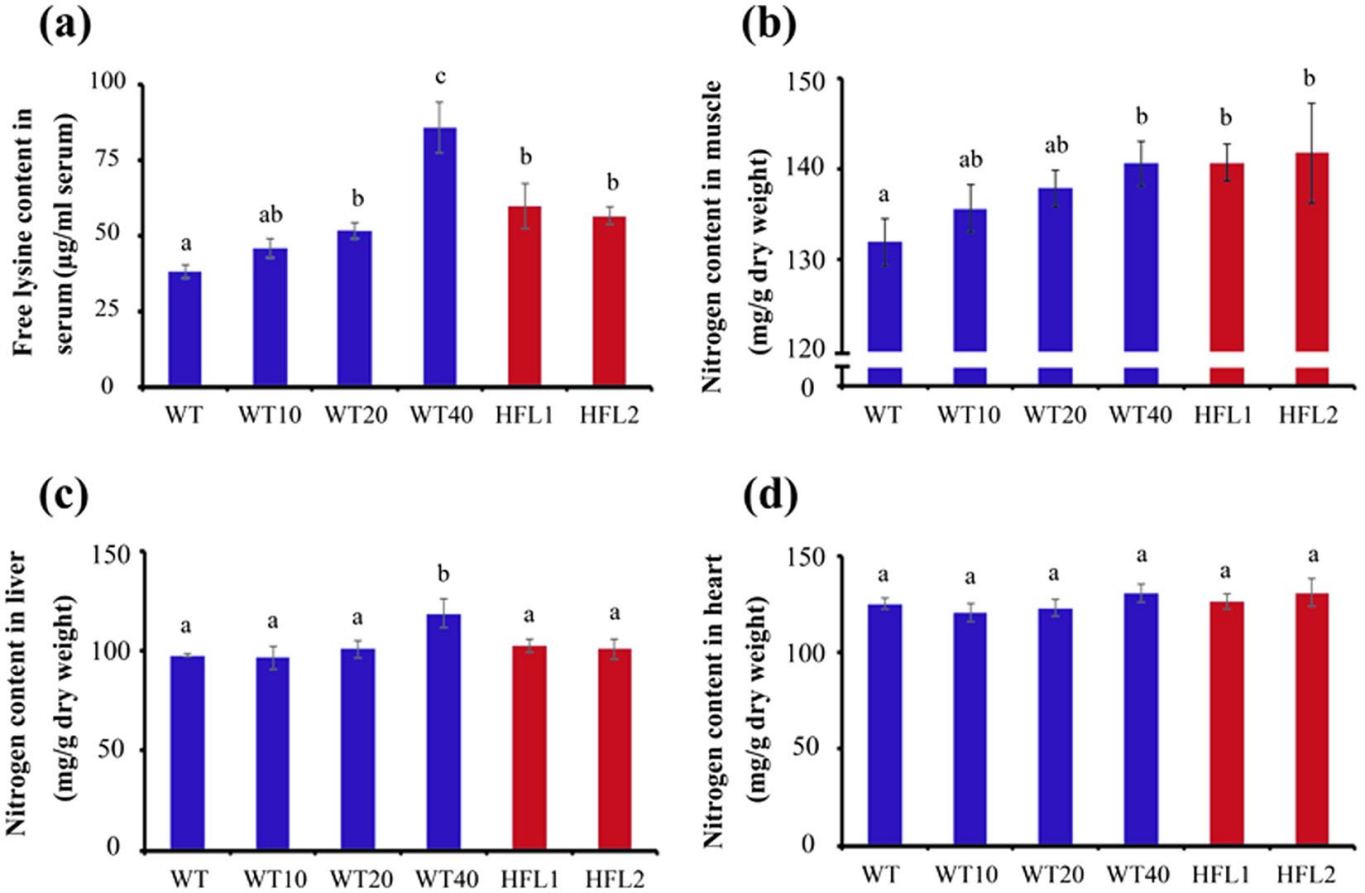
The free lysine content in serum (**a**), nitrogen content in spine muscle (**b**), liver (**c**), and heart (**d**) of rats fed different diets. Error bars represent SD (n = 8). Different letters represent significant differences (*P* < 0.05).

At the end of the feeding experiment, the free lysine in serum and nitrogen in some organs of all groups were measured, and the data were presented in Fig. 6. The results shown that serum lysine content was increased significantly in the HFL groups and WT40 group, with no significant increase in WT10 and WT20 groups, compared with that of WT groups (Fig. 6a). Furthermore, nitrogen content in spine muscle was positively correlated with dietary lysine level (Fig. 6b), while moisture in muscle was not significantly different within different groups (Supplementary Fig. S4). But, there was similar nitrogen level in heart and liver tissues among the six groups, except it was greater in the WT40 group than other groups. (Fig. 6c,d) These data suggested that the serum lysine level and muscle nitrogen content could be improved in growing rats fed either high lysine HFL transgenic rice or wild type rice supplemented with crystalline L-lysine.

### Curve fitting for diets differing in lysine content

**Figure 7:**
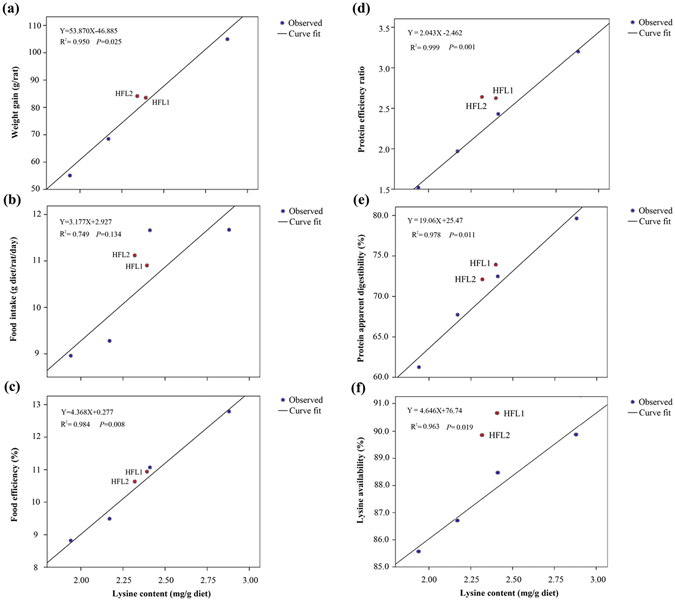
Linear relationship between dietary lysine concentrations and growth biomarkers. The coefficient fitted the value of the linear function. Observed mean values are represented by blue (WT, WT10, WT20, and WT40) and red (HFL1 and HFL2) dots. Solid line represents the fitted dose-response curve. A–F represent the dose-response curve between lysine concentrations and body weight gain (**a**), food intake (**b**), food efficiency (**c**), protein efficiency ratio (**d**), protein apparent digestibility (**e**), and lysine availability (**f**).

All diets in present study, which were derived from the same rice cultivar (same genetic background), differed in lysine content. Thus, we evaluated the relationship between lysine content in the diets and animal growth performance as well as biomarkers (Fig. 7). Animal growth performance parameters (e.g., body weight, food efficiency, protein apparent digestibility, PER, and lysine availability) were higher in WT10, WT20, or WT40 groups than in WT group. The data were subjected to regression analysis to assess the dietary lysine requirement of growing rats. We obtained a linear relationship between lysine concentration and feeding response (Fig. 7). Except for food intake, all other animal growth performance parameters had *R* ^*2*^ values more than 0.95 (*P* < 0.05) (Fig. 7).

Amino acid analyses revealed that lysine concentrations increased by 24% and 19% in HFL1 and HFL2 transgenic rice flour, respectively (Table 2). The biological indicators of rats fed the HFL1 and HFL2 diets might be similar to those of rats fed the WT20 diet, because all three diets had similar lysine concentrations. According to the regression line (Fig. 7e), the theoretical apparent digestibility values of the HFL1 and HFL2 groups were 71.21% and 69.69%, respectively (*R* ^*2*^ = 0.978). Interestingly, the actual apparent digestibility values of the HFL1 and HFL2 groups were 74.01% and 71.98%, respectively, which were slightly higher than the theoretical values. Similar results were also observed with other indexes, including body weight gained, food efficiency, protein efficiency ratio, and lysine availability (Fig. 7).

## Discussion

The two HFL transgenic rice lines had higher lysine concentrations, total protein concentrations, and lysine scores than WT rice^25^ (Supplementary Table S2). The nutritional value of foods depends on the protein amount, protein quality, and amino acid balance^36^. In general, the quality or nutritional value of proteins is dependent on the capacity to replace the nitrogen that the organism inevitably loses during metabolic processes^37^. Therefore, dietary proteins must be evaluated in terms of amount, amino acid profile, and availability^38^. Our findings revealed that the availability of both proteins and amino acids was higher from HFL transgenic rice than from WT rice (Figs 4 and 5).

Several studies have evaluated the nutritional value of transgenic crops, such as rice^39, 40^, maize^41^, soybean^42^, and wheat^43^. In this study, growing rats fed HFL transgenic rice diets had similar growth performance parameters as those fed WT20 diet. The actual protein values and amino acid scores of the HFL groups were within the scope of the theoretical values obtained from curve fittings based on supplemented WT diets (Fig. 7). Therefore, lysine-biofortified rice improved the nutritional value of the crop without affecting other nutrients (Table 2).

Up to now, many studies had shown that the food intake increased in rats fed with diet containing additional lysine, and subsequently the body weight gain, feed efficiency, and growth responsiveness were also improved^26–28^. Indeed, feed intake is usually affected by dietary amino acids^44, 45^. The linkage between dietary lysine to appetite and feed intake had been reported by several groups^46, 47^. The results by Liao *et al*. showed that lysine is a substrate for generating body proteins, peptides, and non-peptide molecules in typical swine diets^46^. Nguyen *et al*. reported that the neuropeptide Y serves as an orexigenic factor^48^. But, these studies suggested further investigation to elucidate the linkage between dietary lysine to arginine ratio and regulation of appetite and food intake. Recently, Liao *et al*. reported that dietary supplementation of crystalline lysine can increase the muscle nitrogen retention and protein accretion, and improved the growth performance of animals^46^.

In this study, the serum lysine content and muscle nitrogen level in rats were also significantly increased in the HFL groups compared with that of WT group (Fig. 6). Both suggested that protein synthesis might be enhanced in rats fed with high lysine diets than those fed WT diet, in agreement with previous reports. Further, in our study, the nitrogen level in other organs (liver and heart) showed no significant difference between the HFL groups and WT group, in agreement with the results of Luo *et al*.^49^, with the exception that the WT40 group rat fed high lysine diet had higher nitrogen content in liver than those fed with WT or WT20 diet (Fig. 6c). This is probably because of liver is regarded as a site for primary pathway of lysine catabolism (saccharopine pathway), which contributes to the whole body lysine catabolism^46, 50, 51^, while supplemental L-lysine level in WT40 diet might be excessive to be digested in time in the growing rats.

The de novo synthesis of amino acids, especially lysine, by gut bacteria takes place in both the small and large intestines^52^. A number of studies have shown that dietary protein including protein source and amino acid composition can greatly influence the gut microbial community^53^. These may be due to decarboxylation of amino acids and peptides leading to the formation of a large array of amines, which are considered to be contributed by the clostridia, bifidobacteria and bacteroides^54^. However, there were also studies on lysine supplementation that indicated lysine as limiting amino acid in the basal diet, but it did not influence the microbial counts in feces and small intestine^55^. In our study, as the main objective was to evaluate the nutritive value of HFL rice on growing rats, we did not measure the gut microflora. It would be interesting to further elucidate the mechanism and function of additional lysine on animal growing.

The quality of food ingredients is estimated by digestibility, availability, and efficiency of amino acids and protein^56^. Among them, amino acid availability is an important factor because amino acids might interact with other components in the diet^57^. O’Quinn *et al*.^26^ reported that digestible lysine increased from 0.80% to 1.15% in young pigs fed high-lysine maize diets. Compared to wild-type lupin seeds, high-lysine and high-methionine transgenic lupin seeds contributed to greater weight gain and higher net protein efficiency and biological value in rats^58^. In this study, lysine availability increased in growing rats fed HFL diets (Fig. 5). Moreover, body weight and PER increased in the groups fed high-lysine transgenic rice diets (Figs 2 and 4). The lysine-biofortified transgenic rice not only contributes to a more cost-effective and sustainable food/feed, but also reduces nitrogen emission levels.

Due to ethical considerations, this study did not include a group of rats fed with diet without protein to provide quantitation of endogenous nitrogen from protein metabolism, thus the protein apparent digestibility was calculated instead of true digestibility. Boisen *et al*.^59^ reported that true digestibility of three raw *Indica* rice diets (7.5 to 10.4% protein) ranged between 68% and 78%. In this study, apparent digestibility was slightly lower (60% to 75%), possibly due to a lower protein content in milled rice than in non-milled rice. Lysine availability ranged between 85% and 90%, which was slightly higher than that reported from high-lysine corn (80%)^60^.

Dietary lysine may affect the metabolism of other nutrients^61, 62, 63^. Excessive dietary lysine has antagonistic effects on arginine in rats, chicks, guinea pigs, and dogs, but not in growing pigs or adult cats^64, 65^. Baker^64^ reported that excessive dietary lysine enhances arginine catabolism by inducing the synthesis of arginase in the kidneys. In pigs, excessive dietary lysine affects growth performance rates^65^. In addition, excessive lysine may affect calcium metabolism, protein methylation, and hormone synthesis in animals^46, 62, 66^. In fish, excessive lysine decreases protein and lysine deposition^67^. In this study, we evaluated the effects of lysine on the absorption of other amino acids. The results revealed that lysine had no obvious effects on arginine, threonine, valine, phenylalanine, or histidine absorption in the WT40 group (Fig. 5 and Supplementary Fig. S3).

The shape and slope of dose-response curves have been used to assess drug doses, relative biological effectiveness, dietary nutrient requirements^67, 68, 69, 70^. In this study, we obtained a linear relationship between lysine concentrations and weight gain, food efficiency, apparent digestibility, PER, and lysine availability (Fig. 7). However, food intake was not affected (*P* > 0.05) by dietary lysine concentrations^71^. The curve fitting results revealed that apparent digestibility, PER, and lysine availability of high-lysine transgenic rice HFL1 and HFL2 were slightly superior to the theoretical values (Fig. 7f). It might due to: (1) The HFL1 and HFL2 contain not only higher lysine content but also more other nutrients, such as total protein and leucine (Table 2 and Supplementary Table S1); (2) The increase level of lysine in biofortified rice gives much higher nutritive value than that in L-lysine-supplemented WT rice. Taken together, the present HFL transgenic rice lines had higher nutritional value not only than the non-transgenic wild type but also wild type rice supplemented with corresponding level of L-lysine (Figs 2–4, Table 2, and Supplementary Table S1). Therefore, our lysine biofortified rice could be further considered for either food or feed application. We are currently conducting food safety assessment of these transgenic rice.

## Methods

### Rice flour preparation

Two high-lysine pyramid transgenic rice lines HFL1 and HFL2 and the non-transgenic wild-type (WT) Wuxiangjing 9 (*Oryza sativa* L. spp. *japonica*) were simultaneously grown at the experimental fields of Yangzhou University (Yangzhou, China) under identical climatic conditions. Seeds were harvested, milled, and processed into flour for composition analyses. For nutrient analyses, three random samples were selected from each type of rice, and each sample was analyzed in triplicate.

### Diet preparation

A total of six rice diets were prepared based on the American Institute of Nutrition AIN-93G formulation (Table 1) for feeding experiments^72^. Two diets, HFL1 and HFL2, were prepared with rice flour from HFL1 and HFL2, respectively. The WT diet contained rice flour from the non-transgenic control WT. The WT10, WT20, and WT40 diets were prepared from WT rice flour supplemented with different concentrations (0.235, 0.47 and 0.94 g/kg, respectively) of crystalline L-lysine (Sigma, USA). L-lysine-supplemented WT rice contained 10% (WT10), 20% (WT20), and 40% (WT40) more lysine than the WT rice flour. Accordingly, HFL1 and HFL2 transgenic rice lines contained about 24% and 19% more total lysine content, respectively, in grains compared with that of the WT rice (Table 2).

All the above six diets contained the same level of mineral, vitamin and fiber. The protein, amino acids and starch were solely derived from rice and lysine supplement, amounting up to 85% (by weight) of the diets (Table 1). The rice diets were vacuum-packed with polyethylene bags until use.

### Animals and experimental design

Animal feeding experiments were carried out in an animal house of the the Chinese University of Hong Kong (CUHK; Hong Kong, China). The experimental procedure was approved by the Animal Experimentation Ethics Committee of CUHK (AEEC No. 14/131/MIS). All experiments were performed in accordance with the guidelines for the use of live animals. In this study, 48 weaned, male Sprague-Dawley (SD) rats (3 weeks of age, body weight at 50 g around) were divided into six groups (with eight rats per group) and fed the corresponding diets (Table 1). Initially, rats were housed individually in polycarbonate cages with stainless steel covers. The room was maintained at 22 ± 2 °C and 45 to 60% relative humidity with 12-h light/dark daily cycles. Following a seven-day acclimation period, rats were housed individually in stainless steel metabolic cages in the same environment, and fecal and urine samples were collected. After six days of nitrogen balance, the rats were transferred to the polycarbonate cages for the end of the experiment.

During the experiments, the rats had ad libitum access to feed and water. Body weight and food intake were recorded daily. Urine and fecal samples were stored at −80 °C. At the end of the experiment, the rats were anaesthetized with sodium barbital and blood was collected through cardiac puncture. Serum was collected from blood samples by centrifugation (3000 x g for 30 min at 4 °C), then stored at −70 °C until analysis. Muscle samples from *Longissimus dorsi*, liver, heart, kidneys, stomach, spleen, lung, and intestine were collected and weighed. All samples were stored at −70 °C until analysis.

### Chemical analysis

Dry matter, ash, fiber, and total fat of rice flours were determined by AOAC methods^73^. Carbohydrate concentration was calculated using the following equation, carbohydrates (%) = 100% − (% protein + % fat + % moisture + % ash)^73^. Crude protein content was estimated by Kjeldahl^74^. Starch and amino acid content were determined as reported by methods we used previously^25, 75^. Moisture in grains and muscle were determined by standard methods^70^. The organ samples were freeze-dried and then ground for nitrogen analyses. The serum was deproteinized using an Ultrafree-MC 10,000 nominal molecular weight limit filter unit (Millipore, Bedford, MA, USA) at 5000 g, 4 °C for 30 min^76^, and the supernatant filtrate was analyzed for amino acids by HPLC analysis^25^.

Feces and urine, which were collected daily during six days of nitrogen balance, were pooled and stored at −80 °C^77, 78^. The samples were freeze-dried and ground for nitrogen and amino acid analyses. The dried urine samples were acidified with 1 mol/L HCl, and the dried fecal samples were hydrolyzed by HCl and subjected to HPLC analysis^25^.

## Calculations

Amino acid score (AAS) was calculated according to WHO recommendations for school-aged children and adolescents^34^. The AAS of sulfur-containing amino acids (methionine and cysteine) was not determined because cysteine was added to the diets. AAS was calculated using the following equation, AAS = amino acid (mg) in 1 g of test protein/reference pattern. The reference pattern of each amino acid is presented in Supplementary Table S3. Food intake (g diet/rat/day) was determined from the average amount of diet consumed by each rat during 70 d. Food efficiency (%)^79^ was calculated as body weight gain (g)/food intake (g) × 100. Apparent protein digestibility of proteins was calculated from (IP - FP) × 100/IP, where IP and FP represent ingested proteins and fecal proteins by weight, respectively. Protein content was calculated using a nitrogen-to-protein conversion factor of 6.25. Protein efficiency ratio (PER) was determined as: PER = (final body weight - initial body weight)/protein intake^80^. Amino acid availability was determined by the method^45, 81^:

$$\begin{array}{l}{\rm{amino}}\,{\rm{acid}}\,{\rm{availability}}( \% )=({\rm{the}}\,{\rm{total}}\,{\rm{intake}}\,{\rm{of}}\,{\rm{amino}}\,{\rm{acid}}-{\rm{fecal}}\,{\rm{excretion}}\,{\rm{of}}\,{\rm{amino}}\,{\rm{acid}})/\\ \,\,\,\,\,\,\,\,\,\,\,\,\,\,\,({\rm{total}}\,{\rm{intake}}\,{\rm{of}}\,{\rm{amino}}\,{\rm{acid}})\times 100.\end{array}$$

### Statistical analysis

Results are presented as mean ± SD. Statistical comparisons were designed to determine whether the differences between the transgenic and WT groups were attributed to lysine content. Homogeneity of variance was determined by one-way analysis of variance (ANOVA) using SPSS 17.0 for windows (SPSS Inc., Chicago, IL, USA). Differences were considered significant at *P* < 0.05. Regression model and curve fitting were used to determine the optimal dietary lysine concentration for growing rats. The relationship between dietary lysine and biological indicators were subjected to regression analysis. Theoretical values were determined from curve fitting.

## Electronic supplementary material

Supplementary Tables and Figures
