# Author Correction: Improved growth performance, food efficiency, and lysine availability in growing rats fed with lysine-biofortified rice

**DOI:** 10.1038/s41598-025-88645-6

**Published:** 2025-02-06

**Authors:** Qing-Qing Yang, Pui Kit Suen, Chang-Quan Zhang, Wan Sheung Mak, Ming-Hong Gu, Qiao-Quan Liu, Samuel Sai-Ming Sun

**Affiliations:** 1 Key Laboratory of Crop Genetics and Physiology of Jiangsu Province/Key Laboratory of Plant Functional Genomics of the Ministry of Education, College of Agriculture, Yangzhou University, Yangzhou, 225009 China; 2 State Key Laboratory of Agrobiotechnology, School of Life Sciences, The Chinese University of Hong Kong, Shatin, Hong Kong, China; 3 Co-Innovation Center for Modern Production Technology of Grain Crops of Jiangsu Province/Joint International Research Laboratory of Agriculture and Agri-Product Safety of the Ministry of Education, Yangzhou University, Yangzhou, 225009 China

Correction to: *Scientific Reports* 10.1038/s41598-017-01555-0, published online 02 May 2017

This Article contains an error in the Discussion section.

“Up to now, many studies had shown that the food intake increased in rats fed with diet containing additional lysine, and subsequently the body weight gain, feed efficiency, and growth responsiveness were also improved^26-28^.”

should read:

“Up to now, many studies had shown that the food intake increased in animals fed with diet containing additional lysine, and subsequently the body weight gain, feed efficiency, and growth responsiveness were also improved^26-28^.”

