# Supplementary material for: Improved growth performance, food efficiency, and lysine availability in growing rats fed with lysine-biofortified rice
Source: Sci Rep. 2017 May 2;7:1389. doi: 10.1038/s41598-017-01555-0 (PMC5430985; doi:10.1038/s41598-017-01555-0)
Supplement: Supplementary file 1 — Supplementary Tables and Figures [file 41598_2017_1555_MOESM1_ESM.pdf]

Research Article

Improved growth performance, food efficiency, and lysine availability in growing rats fed with lysine-biofortified rice

Qing-Qing Yang[1,2], Pui Kit Suen[2], Chang-Quan Zhang[1,3], Wan Sheung Mak[2], Ming-Hong Gu[1], Qiao-Quan Liu[1,3,*] & Samuel Sai-Ming Sun[2,*]

[1]Key Laboratory of Crop Genetics and Physiology of Jiangsu Province / Key Laboratory of Plant Functional Genomics of the Ministry of Education, College of Agriculture, Yangzhou University, Yangzhou 225009, China

[2]State Key Laboratory of Agrobiotechnology, School of Life Sciences, The Chinese University of Hong Kong, Shatin, Hong Kong, China

[3]Co-Innovation Center for Modern Production Technology of Grain Crops of Jiangsu Province / Joint International Research Laboratory of Agriculture and Agri-Product Safety of the Ministry of Education, Yangzhou University, Yangzhou 225009, China

[*]**Correspondences:**

E-mail: qqliu@yzu.edu.cn (Q.Q.L.) and ssun@cuhk.edu.hk (S.S.M.S.).

Supplementary information

Supplementary Tables.

Supplementary Table S1. Amino acid concentrations in HFL and WT diets.

Amino acid (mg/g diet, dry weight)		WT	HFL1	HFL2
Essential amino acids	Lysine	1.94±0.09 a	2.40±0.06 b	2.32±0.14 b
	Histidine	1.25±0.06 a	1.42±0.05 b	1.40±0.12 a
	Isoleucine	2.21±0.08 a	2.39±0.12 a	2.52±0.07 b
	Leucine	3.42±0.12 a	3.68±0.16 a	3.76±0.13 b
	Phenylalanine + tyrosine	4.87±0.30 a	5.37±0.16 a	5.21±0.14 a
	Threonine	2.23±0.13 a	2.14±0.14 a	2.15±0.13 a
	Methionine	2.30±0.01 a	2.61±0.04 b	2.35±0.01 a
	Valine	3.66±0.06 a	4.11±0.12 b	3.98±0.11 b
Non-essential amino acids	Aspartate	5.44±0.39 a	6.42±0.37 b	6.33±0.20 b
	Serine	2.92±0.11 a	2.87±0.36 a	2.91±0.16 a
	Glutamate	10.41±0.24 a	11.26±0.62 a	10.96±0.06 a
	Glycine	2.29±0.08 a	2.52±0.10 b	2.50±0.05 b
	Alanine	3.34±0.11 a	3.92±0.08 b	3.83±0.11 b
	Arginine	4.20±0.72 a	4.31±0.08 a	4.24±0.13 a

Supplementary Table S2. Proposed scores of essential amino acids of different diets (n = 3).

**Amino acid**	**WT**	**HFL1**	**HFL2**	**Reference (g/kg protein)***
Lysine	0.75	0.83	0.78	48
Histidine	1.45	1.47	1.41	16
Isoleucine	1.29	1.24	1.27	30
Leucine	1.01	0.97	0.96	61
Phenylalanine + tyrosine	2.24	2.19	2.07	41
Threonine	1.69	1.64	1.61	25
Valine	1.64	1.63	1.54	40

* The proposed amino acid scoring pattern was based on the WHO 2007 report for school-aged children and adolescents.

Supplementary Table S3. Growth performance parameters of growing rats fed with different diets for 70 d.

Performance	WT	WT10	WT20	WT40	HFL1	HFL2
Initiate body weight (g/rat)	51.34±1.06 a	50.90±1.52 a	51.58±1.22 a	50.81±1.99 a	51.41±1.02 a	50.81±1.16 a
Final body weight (g/rat)	106.44±5.43 a	119.39±12.37 ab	141.86±10.07 cd	155.78±18.30 d	134.08±12.29 bc	134.54±8.04 bc
Body weight gain (g/rat)	55.10±5.25 a	68.49±11.36 ab	90.29±10.31 cd	104.96±18.85 d	82.66±12.11 bc	83.73±8.49 bc
Food intake (g diet/rat/day)	8.96±1.13 a	9.28±0.94 ab	11.66±1.15 b	11.67±1.50 b	10.86±1.21 bc	11.38±1.54 b
Food efficiency (%)	8.82±0.57 a	9.49±0.88 b	11.07±0.82 b	12.79±0.83 c	10.86±0.88 b	10.57±0.79 b

Supplementary Table S4. Organ weight-to-body weight ratio of rats at the end of the feeding trial.

Organ/body (% by weight)	WT	WT10	WT20	WT40	HFL1	HFL2
Liver/body	3.24±0.38 a	3.21±0.59 a	2.95±0.30 ab	2.68±0.25 b	2.88±0.19 ab	2.86±0.13 ab
Kidney/body	1.03±0.05 a	0.96±0.12 ab	0.90±0.03 ab	0.79±0.09 b	0.92±0.05 ab	0.96±0.04 ab
Heart/body	0.47±0.02 a	0.47±0.03 a	0.45±0.03 a	0.44±0.06 a	0.46±0.03 a	0.44±0.04 a
Intestine/body	2.70±0.69 a	2.59±0.90 a	2.49±0.47 a	2.66±1.02 a	2.76±0.95 a	1.61±0.27 a
Spleen/body	0.18±0.02 a	0.18±0.02 a	0.17±0.01 a	0.18±0.02 a	0.18±0.02 a	0.17±0.02 a
Lung/body	0.70±0.09 ab	0.64±0.02 b	0.97±0.31 a	0.67±0.07 ab	0.97±0.39 a	0.74±0.12 ab
Stomach/body	0.97±0.19 a	1.32±0.48 a	0.93±0.20 a	0.77±0.12 a	0.98±0.20 a	0.85±0.22 a

Supplementary Figures

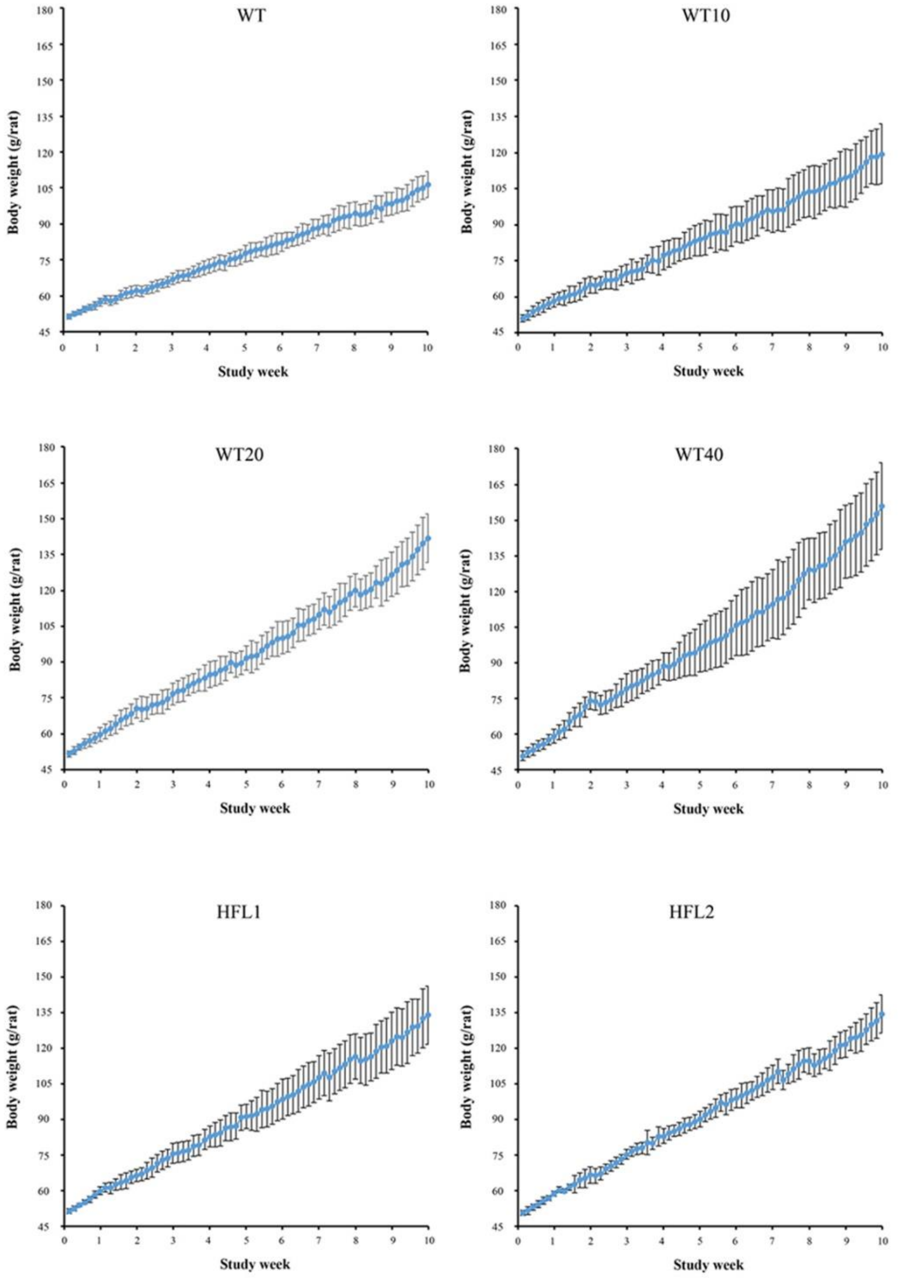

Supplementary Figure S1. Body weight (with SD for each point) changes in rats fed different diets for 70 d.

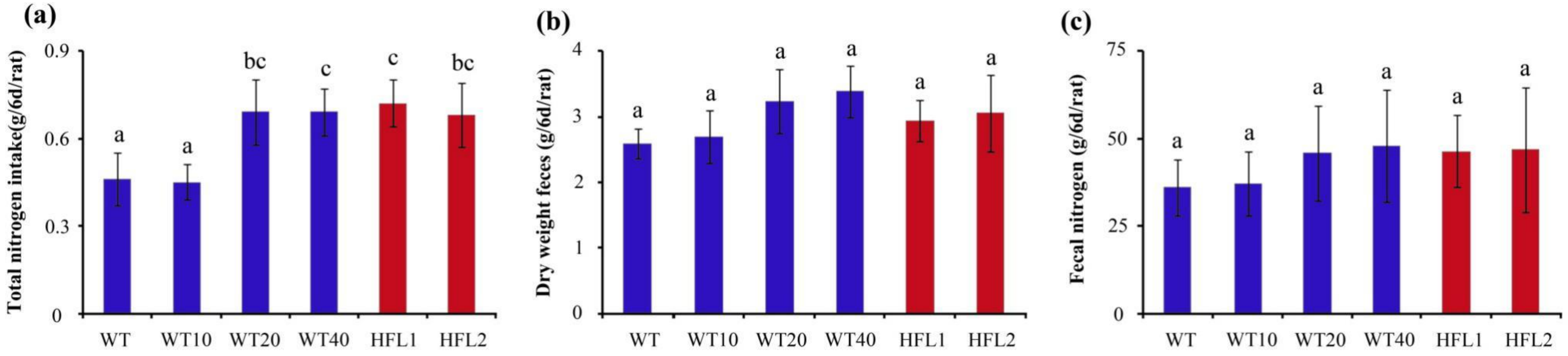

Supplementary Figure S2. Total nitrogen intake (a), and fecal (b) and urinary (c) excretion of different groups during the nitrogen-balance stage. Error bars represent SD (n = 8). Different letters represent significant differences ($P < 0.05$).

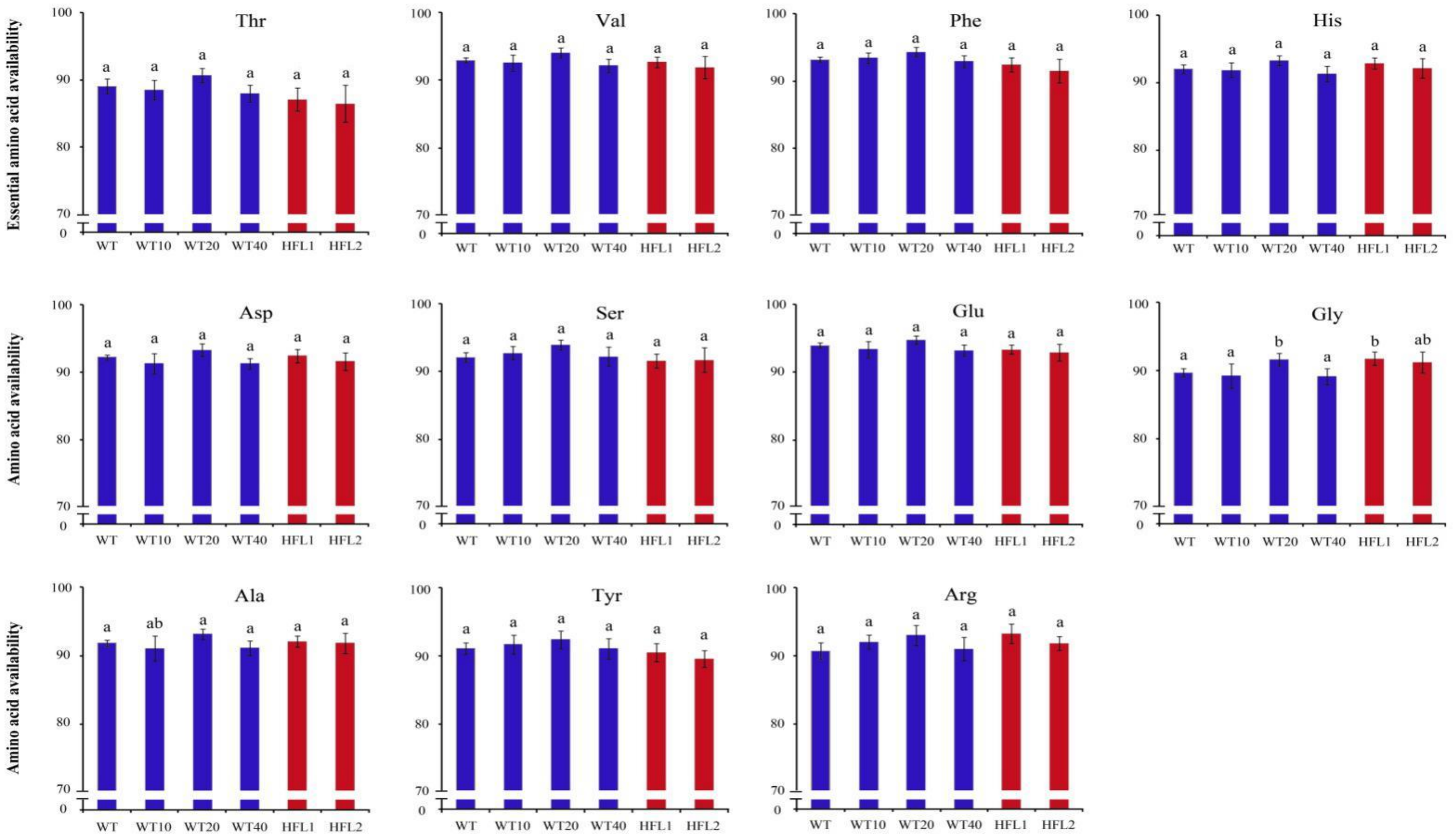

Supplementary Figure S3. Availability of other amino acids in rats fed different diets. Error bars represent SD (n = 8). Different letters represent significant differences ($P < 0.05$).

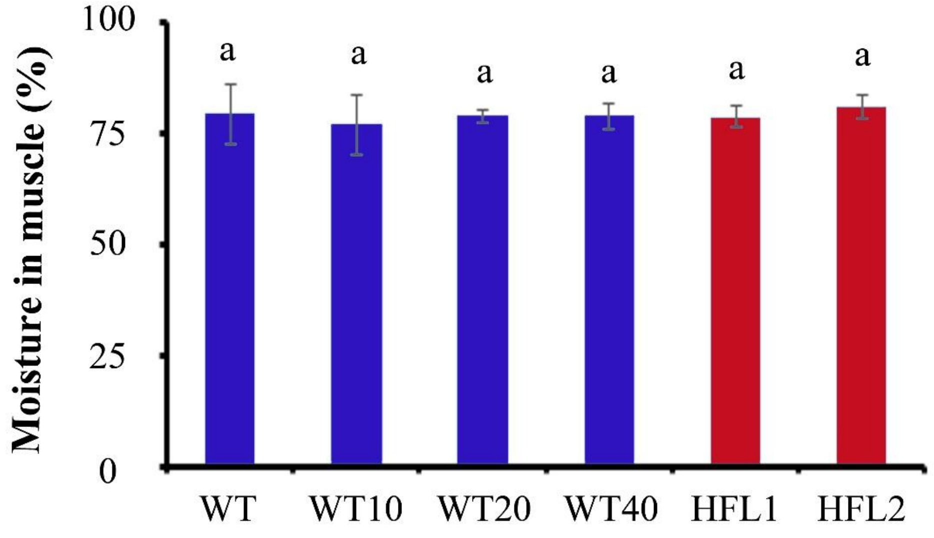

Supplementary Figure S4. The moisture content in spine muscle of the rats fed different diets. Error bars represent SD (n = 8). Different letters represent significant differences (P < 0.05).
